# Quantifying Process-Based Mitigation Strategies in Historical Context: Separating Multiple Cumulative Effects on River Meander Migration

**DOI:** 10.1371/journal.pone.0099736

**Published:** 2014-06-25

**Authors:** Alexander K. Fremier, Evan H. Girvetz, Steven E. Greco, Eric W. Larsen

**Affiliations:** 1 School of the Environment, Washington State University, Pullman, Washington, United States of America; 2 International Center for Tropical Agriculture, Nairobi, Kenya; 3 School of Environmental and Forest Sciences, University of Washington, Seattle, Washington, United States of America; 4 Department of Human Ecology, University of California Davis, Davis, California, United States of America; University of Sydney, Australia

## Abstract

Environmental legislation in the US (i.e. NEPA) requires defining baseline conditions on current rather than historical ecosystem conditions. For ecosystems with long histories of multiple environmental impacts, this baseline method can subsequently lead to a significantly altered environment; this has been termed a ‘sliding baseline’. In river systems, cumulative effects caused by flow regulation, channel revetment and riparian vegetation removal significantly impact floodplain ecosystems by altering channel dynamics and precluding subsequent ecosystem processes, such as primary succession. To quantify these impacts on floodplain development processes, we used a model of river channel meander migration to illustrate the degree to which flow regulation and riprap impact migration rates, independently and synergistically, on the Sacramento River in California, USA. From pre-dam conditions, the cumulative effect of flow regulation alone on channel migration is a reduction by 38%, and 42–44% with four proposed water diversion project scenarios. In terms of depositional area, the proposed water project would reduce channel migration 51–71 ha in 130 years without current riprap in place, and 17–25 ha with riprap. Our results illustrate the utility of a modeling approach for quantifying cumulative impacts. Model-based quantification of environmental impacts allow scientists to separate cumulative and synergistic effects to analytically define mitigation measures. Additionally, by selecting an ecosystem process that is affected by multiple impacts, it is possible to consider process-based mitigation scenarios, such as the removal of riprap, to allow meander migration and create new floodplains and allow for riparian vegetation recruitment.

## Introduction

Society's changing perceptions of “impact” to the natural environment influences how natural areas are protected and how project effects are mitigated. In the United States, The National Environmental Policy Act of 1969 (42 U.S.C. §§4321–4370(a) requires evaluation of impact based on ‘current condition’ [1]; that is, impacts of proposed projects are compared to the current state or baseline of the system and does not include pre-existing alterations. Without properly addressing these cumulative effects over time, environments with a long history of human alteration will incrementally lose natural attributes and move closer to a more completely human-dominated landscape that lacks the structure or function to support natural processes; this phenomenon has been termed a “sliding baseline” [2], [3].

Although cumulative effects are being addressed by the Council on Environmental Quality [4], [5], a standardized protocol for quantitatively assessing them remains elusive [6], [7]. Assessment of cumulative effects therefore requires accurate quantification of past impacts from multiple drivers, anthropogenic or not. Quantifying the legacy of anthropogenic alterations is a significant challenge given natural inter-annual variability and synergisms between multiple forms of impacts [8], [9]. Cumulative effects assessment therefore requires a longer term view of environmental impact and typically evaluates changes to ecosystem processes, not just structures that are impacted [10].

In many cases, ecosystem processes (e.g. fire, flooding, climate, vegetation succession, and habitat connectivity) cannot be fully restored because either historical conditions no longer exist (climate regime or flow regulation due to dams) or the political motivation for alternative scenarios is lacking (risk and monetary considerations). Yet, if we are to avoid a sliding baseline, we must consider the history of changes to individual processes and search for efficient ways to mitigate the effect. This is particularly the case in disturbance-dependent systems, such as riverine-riparian areas, where watershed and river channel alterations influence downstream ecosystem processes and functioning [11], [12]. For these disturbance-dependent ecological communities, alterations to the processes that create and maintain them can have significant and lasting impact [13], such as wetland loss on water on landscape functions [9] or flow regulation effects on river channel migration and riparian vegetation [14], [15].

Quantifying the effects of individual projects on large scale ecosystem processes presents a considerable scientific challenge [6]. Assessment of multiple forms of alterations requires a modeling scientific approach [16]. Where empirical studies are necessary to show impact, retrospective and prospective modeling approaches allows for individual processes and impacts to be separated and quantified. In addition, it allows for development of alternative process-based solutions.

A long-term process-based view is important for understanding riparian vegetation dynamics, restoration and mitigation needs [17]. Natural disturbance processes counteract autogenic forces that drive riparian forest stands into later successional stages through floodplain inundation and by continuously reworking and depositing new lands [18], [19]. Therefore, a static view of ecosystem structure and function is insufficient to maintain pre-impact habitat heterogeneity because individual stands are constantly maturing and the recruitment of new seedlings is necessary to take the place of the aging forest [20]. Conservation of riparian vegetation communities requires fostering both flows those that create new land, by channel avulsion or lateral bend migration [21], and secondly, flows that allow riparian recruitment [22], [23]. In addition, management of patch dynamics is a critically important concept to create and maintain patches suitable to endangered species on rivers [24].

The overall goal of this research was to use a process-based modeling approach to isolate the specific impacts to lateral river channel migration of flow regulation – past and proposed, and current channel constraints (riprap). Using a mechanism-based numerical model of meander migration allowed us to tease apart the relative effects of riprap and flow regulation separately, as well as the interacting effect of the two together. We discuss our results from a sliding baseline perspective, and identify implications of synergism between impacts, the role of modeling in cumulative impact assessment, and possible process-based mitigation solutions.

## Methods

### Study area

**Figure 1:**
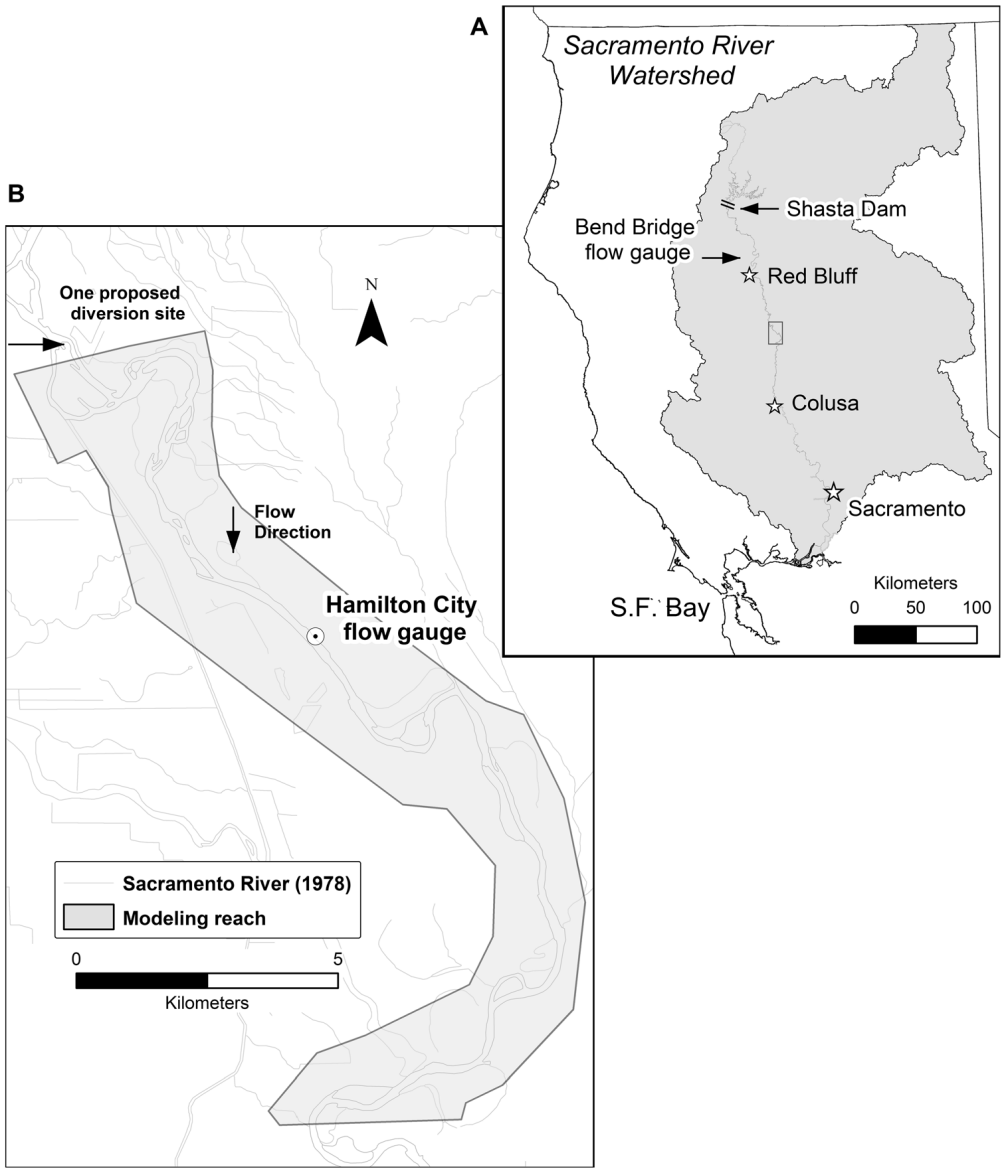
Study site map showing the modeled reach and the historic mapping extent. The meandering reach of the Sacramento River lies between the towns of Red Bluff and Colusa.

The Sacramento River flows south through the Central Valley of California, USA (Figure 1a), which has a Mediterranean climate. The main sources of water come from the Sierra Nevada, Coast and Cascade mountain ranges during winter precipitation and spring snow melt events (Nov-April); it is a semi-arid system with a long dry season between May and October. The river channel is partially free to migrate between the towns Red Bluff and Colusa. The study reach was defined from river miles 184–201(river miles or “RM” as location markers defined by the USACE; 30 km in length) (Figure 1b) to investigate the effect of flow changes. This segment was chosen due to the availability of historical river channel records and management interests. Within the study reach, the Sacramento River is primarily a single-thread sinuous channel. Channel cutoff events are infrequent though present with the reach, occurring on average less than once every two years and account for less than 10% of the newly created floodplain [25]. The riverbed material is sand and pebbly gravel with a median grain size that ranges from 20 to 30 mm; the channel banks are composed of sand and gravel with isolated patches of erosion-resistant rock types [26], [27], [28].

The major man-made structures that have affected the Sacramento River's hydrology in the last 70 years are the Shasta Dam (1943 flow alteration, 1945 completion of construction), located approximately 190 river kilometers upstream from the study site; major flow diversion structures for agriculture (such as the Red Bluff Diversion Dam and the GCID diversion facility); and a number of flood control structures allowing overflow during large flow events into catchment basins and bypass channels. Flow regulation has changed the hydrograph primarily in two ways: 1) winter peak flows were reduced to decrease downstream flooding and increase water storage and 2) summer base flows were increased for dry season agricultural irrigation purposes [29]. Extensive riprap was constructed along the channel margins starting from 1940 up to the present; however, most of the rock revetment in place today was installed between 1970 and 1980 [28]. Approximately one-third of the reach from RM 143.5–194 is constrained by revetment [28]. We selected a model calibration period (1942–1976) and a study reach to avoid calibrating the model in an area with extensive riprap. After dam construction, the valley floor and riparian areas were rapidly cleared and converted to row crops and orchards; and today, 11% of the historic riparian forests remain and only 6% in a non-degraded state [29], [30].

### Stream hydrology reconstruction

Eight flow datasets were constructed and used in the meander migration model (Table 1). One flow record was created to calibrate the erosion field for the meander model between 1942–1976 at the Hamilton City gauge (Table 1, Figure 1a). Two historical flow records were used to represent the pre-and post-dam flows; these records were built from the USGS Bend Bridge (1892-present) and Hamilton City gauges (1945–1976) (Figure 1a). Simple linear regressions were applied to model historical trends and the relationship between the two gauging stations. Flows at the Hamilton City gauge better represent the actual flows at the study reach, while the Bend Bridge record is the longest record for the Middle Sacramento River. The regression analysis between the Bend Bridge and Hamilton City gauges show a strong correlation at both the daily (R^2^ = 0.90, p<0.0001) and yearly time steps (R^2^ = 0.99, p<0.0001).

**Table 1:** Description of flow records (daily mean discharge) measured at USGS Bend Bridge Gauge #1137100 and Hamilton City #11388500, and five simulated flows using the CALSIM model.

	Flow Dates	Description
**Historic Flows (3)**		
** Calibration**	1942–1976	Observed flows for Hamilton City, 1945–1976. From 1942–1944, flows were modeled based on a regression relationship between Hamilton City and Bend Bridge.
** Historical (** ***pre-dam*** **)**	1892–1942	Predicted flows using the historical record at Bend Bridge using the Hamilton City regression relation for pre-Shasta dam time period.
** Historical (** ***post-dam*** **)**	1943–1992	Predicted flows using the historical record at Bend Bridge using the Hamilton City regression relation for post-Shasta dam time period. These records include the effects of additional infrastructure added over time.
**CALSIM II Simulated Flows (5)**		
** Current (** ***base*** **)**	1922–1992	Modeled flow record with all current diversion contracts in place. Based on historical inputs at Bend Bridge gauge, but simulates entire record as if the current diversions had been in place for the entire time.
** NODOS flow scenarios (4): ** ***4a,5b,6 7***	1922–1992	Modeled flow records with all current diversion contracts and NODOS diversions in place. The four scenarios are based on historical inputs at Bend Bridge gauge and simulate different flow management options in the timing and amount of diversions.

The five daily flow management scenarios were simulated using the computer program CALSIM II, as part of the North of Delta Off-stream Storage (NODOS) project, a large planning effort by the California Department of Water Resources (CDWR). CALSIM II is a water resources simulation model designed to evaluate operational alternatives of large, complex river basins. Here, the NODOS project flow scenarios were modeled using quantified knowledge of the entire system. For example, a certain amount of water is necessary year-round both for biological and water quality issues in the upper valley and delta waterways. These scenarios are simulations of the daily river flows that would occur if all current dams and diversions were in operation and are based on the historic record of flows, for the water years 1922–1994.

The NODOS project base hydrograph is a simulation of river flows that would occur under current conditions and is based on flows over the past 70 years. The four scenarios – labeled 4a, 5b, 6, and 7 – are simulations of the flows that would occur with the operation of the NODOS project reservoir and current infrastructure (i.e. management at Shasta Dam and other water diversions and inputs). The four scenarios differ in amount and timing of when water is diverted out of the main channel of the river.

For each hydrology dataset the annual cumulative effective stream power (Ω_ce_) was calculated as a part of the river meander migration model. This parameter has been shown to be proportional to bank erosion [14], [31]. Stream power is used by geomorphologists to quantify the forces that lead to bed and bank movement. Cumulative effective stream power is the summation of stream power over a given threshold value within a given time step (e.g. annually).
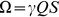
(1)

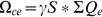
(2)where, stream power (Ω kg m/s^3^) is calculated as the product of discharge (Q m^3^/s), slope (S m/m), and the specific weight of water (γ kg/m^2^s^2^). The effective discharges (Q_e_) are those flows over a determined threshold. The threshold used in this study was determined using historical data and regression analysis [31]. To analyze the cumulative effects of reduced flows we increased the span of the simulation from 70 to 130 years, the approximate time of the flow record at the Bend Bridge gauge.

In addition, in order to test for long-term changes in climate over the model time frame we compiled historical annual average precipitation data (PRISM) of the Sacramento River from 1895–2002. Long-term climatic influences were not apparent as we observed no significant decrease in precipitation.

### River channel meander migration modeling

The river channel meander migration model is a numeric model based on relationships between sediment transport and fluid flow, and has been used to calculate how an alluvial river channel moves over time-scales of years to decades [32], [33]. The model assumes that the local bank erosion rate is proportional to a local water velocity factor such that:
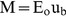
(3)where M is the bank erosion rate (m/s), E_o_ is a dimensionless bank erodibility coefficient of the order 10^−8^, and u_b_ (m/s) is a velocity factor equal to the difference between the velocity near the bank and the reach-average velocity [32]. Higher E_o_ values result in greater erosion potential. Although the model analytically calculates the velocity field in some detail, it represents bank erodibility by an estimated coefficient (E_o_) that is calibrated to observed data.

The erodibility potential for the meander migration model was developed in geographic information system (GIS) based on observed geology and vegetation surfaces and known riprap (bank revetment), all of which vary spatially throughout the erosion field. The geology surface dataset was obtained from the California Department of Water Resources [27]. The geology dataset was used to determine areas that were non-erodible due to geologic constraint. The vegetation dataset, used to distinguish between agricultural and riparian land cover, was derived from aerial photography taken in 1997 [34]. Agricultural lands were coded to be twice as erodible as riparian vegetation based on the work of Micheli et al. [35] within the same section of river. These values provided the initial erosion field, which was subsequently adjusted by calibrating the modeled bank erosion to observed data.

To calibrate the erodibility coefficient to observed conditions, simulations require a calibration process. The calibration period for this study used historical channel position data and flow records from 1942 and 1976 (post dam and prior to major channel constraints). Model parameters were adjusted until model erosion rates matched the observed erosion rates between these time periods. The same parameters were then used for each model run of each scenario (Table 2).

**Table 2:** Meander migration model – geomorphic and hydraulic variables.

Input variable	Description	Value
Discharge (Q_2_)	2-year recurrence interval discharge.	2265 m^3^/sec
Width (H_2_)	Width at Q_2_.	235 m
Depth (W_2_)	Average depth at Q_2_	5.4 m
Slope (S)	Longitudinal water surface slope.	0.00042 m/m
Grain Size (D_50_)	Median bed particle size.	25 mm
Lower threshold	Calculated flow over which significant bank erosion is initiated (Larsen et al. 2006b).	425 m^3^/sec
Recurrence Interval	Flow recurrence interval over which cutoff algorithm triggers potential cutoff (Larsen et al. 2006c).	1.5
Sinuosity Threshold	Channel sinuosity threshold used to trigger cutoff simulation. Calculated for each bend between inflection points (Larsen et al. 2006c).	1.9 m/m

The effect of riprap was simulated by modifying the erosion potential raster surface, using a GIS data layer of riprap occurrence from the US Army Corps of Engineers [36]. The spatial error associated with geo-referencing was less than 30 meters; therefore, a 30 meter cell size (0.09 ha) was used as the spatial resolution for the raster surface. The riprap layer was combined with the erosion potential raster surface to simulate areas into which the channel could not erode (ESRI Version 9.1). Migration patterns with channel revetment (i.e. riprap) were modeled separately from the migration with the flow scenarios in order to separate the effect of flow and channel constraints on bank erosion.

**Figure 2:**
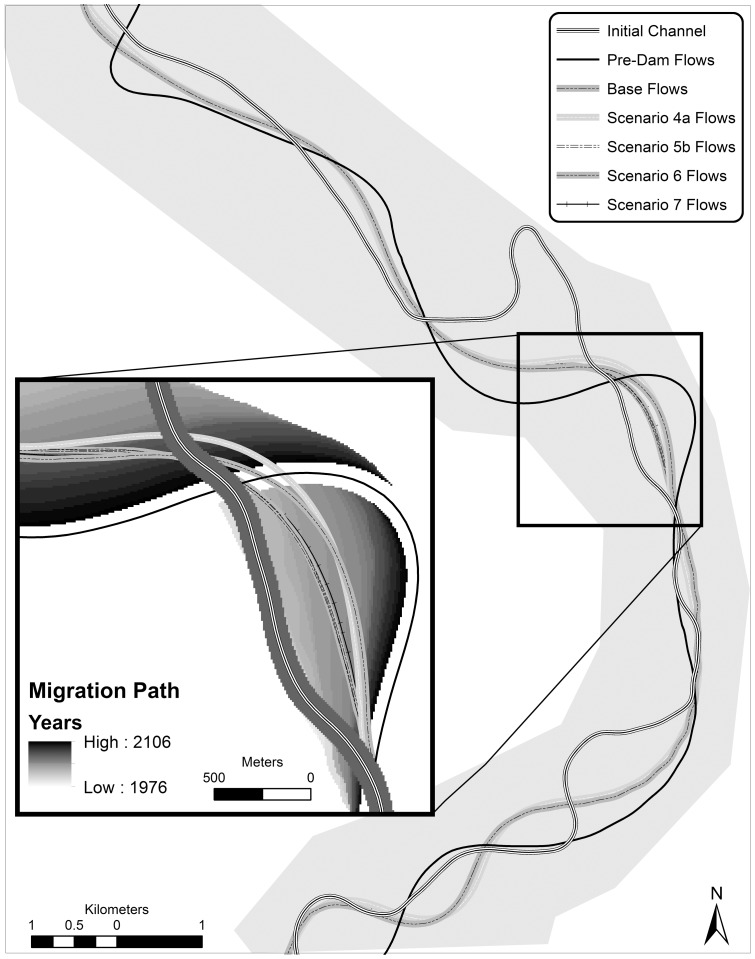
Meander migration model output centerlines for each scenario after 130 year simulation, including the initial channel centerline. The inset map includes a zoomed in migration path of the pre-dam flow channels. Scenarios 5b and 6 are close to overlapping.

To quantify migration patterns, we calculate the ‘area reworked’ (equivalent to ‘depositional area’) by lateral channel migration and channel abandonment. We also report migration rates for comparisons with other studies. Migration rates were calculated by dividing the total area reworked by the average stream length of the first and last year and the simulation time (130 years). This represents the average annual lateral movement of the channel per meter of length. In our modeling runs, one cutoff occurred in the first years of each model run. We did not include this cutoff event in our calculations as it was approximately the same for each scenario (Figure 2).

We also present area reworked by individual bend to illustrate the potential number of bends that would be required to mitigate flow and riprap alterations. Using the GIS we separated the modeled migration area by intersecting the first and last centerline and removing polygons created by cutoff events. We show individual bend migration area for each of the NODOS project base scenarios.

## Results

**Figure 3:**
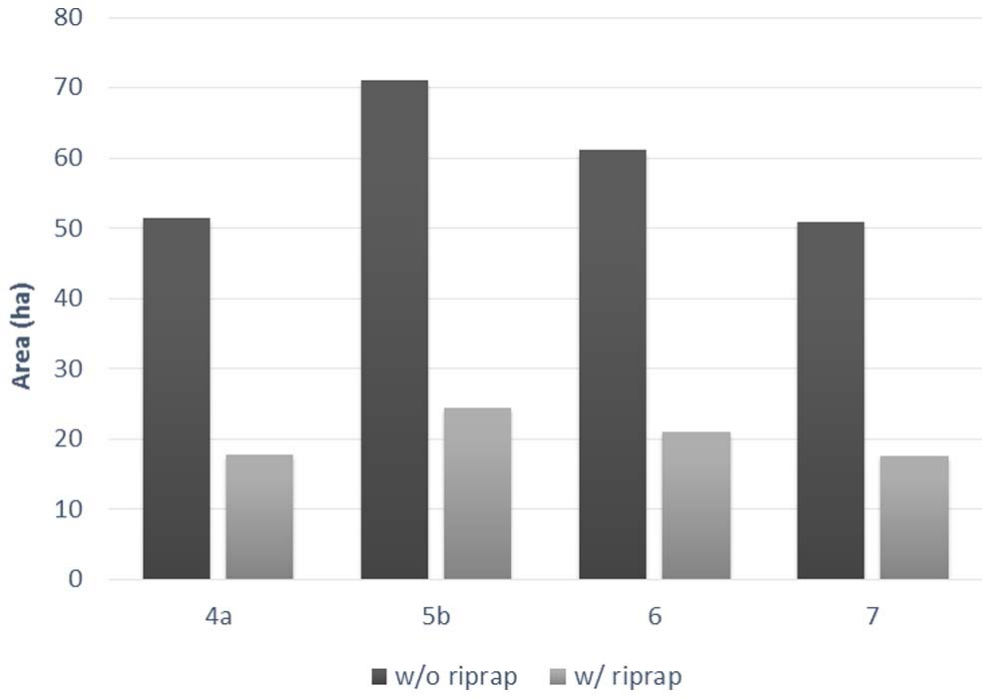
Predicted floodplain reworked by lateral channel migration lost to flow regulation (NODOS scenario flows compared to NODOS base flow) and riprap. These areas represent the area of potential mitigation for the NODOS project. Riprap reduced almost three times (2.89) the area compared to the proposed NODOS flow regulation.

Flow regulation and riprap on the Middle Sacramento River have greatly reduced river meander migration potential (Table 3, Figures 2 and 3). Using pre-dam flows, lateral river channel migration reworked 1103 ha of land over the 130 year modeling period without riprap, compared to 832 ha with post-dam flows. This is a 25% loss in migration potential due only to changes in stream power caused by flow regulation to date; this accounting does not include changes in riparian vegetation, riprap or sediment reduction.

**Table 3:** Comparison of area reworked by river channel migration for seven flow scenarios (see Table 1) with and without riprap over a 130-year period.

Modeled scenarios	Area reworked (ha)	% Diff in area from pre dam	Potential Mitigation (ha)
**No Riprap**			
Pre-dam flow	1103.4		
Post-dam flow	832.4	24.6	
NODOS base flow	687.7	37.7	
flow 4a	636.3	42.3	51.4
flow 5b	616.7	44.1	71.0
flow 6	626.6	43.2	61.1
flow 7	636.8	42.3	50.9
**Current Riprap**			
NODOS base flow	237.3	78.5	
flow 4a	221.6	80.1	17.7
flow 5b	215.9	80.7	24.5
flow 6	218.3	80.4	21.1
flow 7	223.0	80.1	17.6

The numbers in the Potential Mitigation column are the differences between NODOS base and NODOS scenarios. This can be seen as the total area needed to be mitigated if you assume rip-rap absent (top) or rip-rap present (bottom). The percent difference is the mitigation needs if you assume rip-rap present versus absent (289%). The bottom line is that without rip-rap, almost three times (2.89) increase in the area needed to be mitigated (masked effect).

Using the CALSIM simulations for current flows and the proposed NODOS project flows, shows a further decrease in area reworked to 687 ha within the same 130 year period. Cumulatively, the current flows have reduced lateral channel migration 38% compared to pre-dam conditions (Table 3, Figure 3). The proposed NODOS project flows would reduce that further to 42–44%. Compared to current conditions, the proposed NODOS project flows would reduce channel migration by 7–10% as compared to the base NODOS project scenario (Table 3, Figure 3).

### Synergistic effects of flow regulation and riprap

When riprap is modeled with flow regulation, the NODOS project base flow condition shows a 79% cumulative reduction in lateral river channel migration compared to the pre-dam conditions. The percent difference between the NODOS project flow scenario with and without riprap is minimal; however, in absolute terms, the area reworked tells a different story. The NODOS project flow scenarios would reduce the area of floodplain reworked by 51–71 ha without current riprap in place and 17–25 ha with riprap. (Table 3, Figure 3). This difference in modeled impact is almost three times greater (2.9) in absolute area than when channel migration is modeled with riprap in place. Considering mitigation, the effect of riprap, in this case, essentially masks the impacts of flow regulation in absolute area terms. To give perspective to these respective areas, we quantified the area eroded by individual bend. In our 30 km study reach, there were 13 active bends with an average area reworked of 52 ha of land for the NODOS project base flow scenario. This suggests that the removal of riprap and subsequent meander migration from a single bend would approximately mitigate the impact of the proposed NODOS project flow regulation.

## Discussion

In this study we applied a modeling tool to compare and help us separate the effects of flow regulation versus channel revetment on lateral river channel migration. If only empirical data were used, the synergistic interacting effect of altered floodplains (e.g. channel constraints and vegetation clearing) and flow regulation taken together would not have been quantifiable, but see Duinker and Greig 2007 for another example [37]. Our modeling procedure allowed us to quantify the impact of channel revetment from flow regulation in an effort to inform management and restoration of an important large-scale ecosystem processes [38]. Without this process-based modeling approach we would have underestimated the mitigation needs by almost a factor of three (see Table 3).

### Cumulative effects

Considering cumulative impacts, although the NODOS project would reduce channel migration potential 7–10% from the current base scenario, the changes in cumulative effective stream power would have reduced the migration rate 38% when compared to historical pre-dam conditions. The NODOS water diversion project adds to an overall impact of 40–43% from pre-dam conditions, and 79% including flow alterations and riprap. In this case, the cumulative impacts to channel migration on the Sacramento River are considerable relative to the impact of the proposed flow regulation scenarios (Figure 2 and 3).

Deviations from historical baseline condition, such as the ones described here, are a result of cumulative impacts and are not typically included in the assessment of new projects because of difficulties in defining the appropriate spatial and temporal scale, and identifying the history of direct and/or indirect effects [7]. The presence of multiple interacting human and environmental stressors can act synergistically or additively depending on the environmental setting and character of the alteration [8], [12]. Although we might not be able to precisely define the true ‘pristine’ environment, certainly quantified knowledge of some historical impacts will help in ultimately what we aim to do, which is to assess impacts to the function and structure of the riverine-riparian ecosystem. As illustrated in this study, when the baseline conditions are defined as the system's current conditions and the system has a long history of human alteration, the environment slides farther from historical conditions. Without properly addressing the cumulative effects over time, environments with a long history of human alteration will incrementally lose natural attributes and move closer to a more completely human-dominated landscape that lacks the structure or function to support ecosystem processes.

### A process-based mitigation approach

Using a meander migration model we were able to isolate a plausible ecosystem process-based mitigation scenario for the proposed off-stream storage facility. Our results suggest that there are enough geomorphically significant flows to move the channel laterally in the absence of near-channel constraints (riprap), albeit less dynamic and smaller in extent than historical pre-dam conditions [14], [31]. One potential management action is targeted removal of channel constraints (riprap) to mitigate the effect of additional water diversions and withdrawals. In our study, the removal of riprap on a single bend would generate enough area to mitigate the impact of the proposed water diversion, assuming lateral channel migration is possible at the site (see Figure 2).

Our goal was to investigate the long-term direction of a single large-scale driver of ecosystem change (i.e. a keystone process), river channel migration. We aimed to isolate the impact of a first-order control, hydrology, on channel migration by holding all else equal. On this river reach, summer base flows (typically less than 425 m^3^/s) were found not to cause significant lateral migration through the initiation of bank erosion [15]. Flow scenarios including this lower threshold will show increased impact from flow regulation and diversion because of the redistribution of flows from a few large peak events to many days of flows below the threshold, i.e. dry-season base flows. Without consideration of the lower threshold, the effect of the altered flow magnitude would be missed entirely. Including this geomorphic threshold in the planning process of dam re-operation would be a valuable way to account for the impact of proposed flow management on this vital ecosystem process [39].

Our analysis of system behavior is the beginning of more specific models to quantify the impact of sediment supply loss, riparian vegetation controls on bank erosion rates, adjustments in channel valley slope, and specific riparian vegetation recruitment mechanisms. In selecting the model procedure and parameters, we aimed to minimize model complexity and isolate the impacts of one flow parameter (cumulative effective stream power) on floodplain development. Our study did not model changes in sediment supply due to dam construction which is important to consider as multiple studies have reported reduced lateral migration rates post-impoundment [40]. In addition, to focus the paper, we also excluded a detailed analysis of cutoff dynamics within the reach that might be impacted by flow regulation [16], [26]. A more detailed study into flow effects on cutoff dynamics would improve our process-level understanding channel movement in large rivers [41].

Riparian forest extent, pattern and structure have important implications for channel migration rate and bend shape [41], [42]. In this study we did not alter the riparian vegetation pattern between scenarios, though we used GIS data to represent the impact of vegetation on bank resistance by increasing bank resistance two-fold between agriculture and riparian vegetation [41]. Previous historical work on lateral channel migration on the Sacramento River suggests that riparian vegetation might have had a large impact on erosion rates post flow regulation [21]. We excluded riparian vegetation in our scenarios, in part, to limit the complexity of the scenarios, but also because it was difficult to envision a management scenario where vegetation would be removed to increase lateral channel migration. Riparian vegetation in the reach is actively being restored to meet multiple conservation objectives [29] due to the legacy of riparian forest loss in this river system.

### Summary

Channel migration is an important ecosystem process on low-gradient rivers that structures floodplain environments. We employed a mechanism-based model of river channel migration to quantify the impacts of past, current and proposed flow regulation on lateral river channel migration patterns. We found that channel migration potential would be further reduced with the proposed off-stream storage facility (7–9%), adding to previous flow regulation caused by impoundments upstream (38%). Riprap further reduced channel migration (79%) compared to historical flow patterns. Given competing needs in the state for water, we describe a potential solution to limit conflicts by removing enough riprap to allow lateral migration to occur and offset the impacts of flow regulation. This potential solution focuses on the processes that create and maintain ecosystems and is potentially a quantifiable way to design new approaches to mitigate impacts among multiple stressors in systems with extensive cumulative impacts.
